# Cost‐effectiveness of scaling up a whole‐of‐community intervention: The Romp & Chomp early childhood obesity prevention intervention

**DOI:** 10.1111/ijpo.12915

**Published:** 2022-03-17

**Authors:** Huong Ngoc Quynh Tran, Anagha Killedar, Eng Joo Tan, Marj Moodie, Alison Hayes, Boyd Swinburn, Melanie Nichols, Vicki Brown

**Affiliations:** ^1^ Deakin Health Economics, Institute for Health Transformation, School of Health and Social Development, Deakin University Geelong Victoria Australia; ^2^ Global Obesity Centre (GLOBE), Institute for Health Transformation, School of Health and Social Development, Deakin University Geelong Victoria Australia; ^3^ Faculty of Medicine and Health, School of Public Health, The University of Sydney Sydney New South Wales Australia; ^4^ School of Population Health, University of Auckland Auckland New Zealand

**Keywords:** childhood obesity, cost‐effectiveness, economic evaluation, prevention

## Abstract

**Background:**

Given the high prevalence of early childhood overweight and obesity, more evidence is required to better understand the cost‐effectiveness of community‐wide interventions targeting obesity prevention in children aged 0–5 years.

**Objectives:**

To assess the cost‐effectiveness of the Romp & Chomp community‐wide early childhood obesity prevention intervention if delivered across Australia in 2018 from a funder perspective, against a no‐intervention comparator.

**Methods:**

Intervention costs were estimated in 2018 Australian dollars. The annual Early Prevention of Obesity in Childhood micro‐simulation model estimated body mass index (BMI) trajectories to age 15 years, based on end of trial data at age 3.5 years. Results from modelled cost‐effectiveness analyses were presented as incremental cost‐effectiveness ratios (ICERs): cost per BMI unit avoided, and cost per quality‐adjusted life year (QALY) gained at age 15 years.

**Results:**

All Australian children aged 0–5 years (*n* = 1 906 075) would receive the intervention. Total estimated intervention cost and annual cost per participant were AUD178 million and AUD93, respectively, if implemented nationally. The ICERs were AUD1 126 per BMI unit avoided and AUD26 399 per QALY gained (64% probability of being cost‐effective measured against a AUD50 000 per QALY threshold).

**Conclusions:**

Romp & Chomp has a fair probability of being cost‐effective if delivered at scale.

**Abbreviations**
“B” cohort: The LSAC baby cohort
“K” cohort: The LSAC kindergarten cohort
APPLE: a pilot program for lifestyle and exercise
AUD: Australian dollar
BAEW: be active eat well
BMI: body mass index
CEA: cost‐effectiveness analysis
CHAT: communicating healthy beginnings advice by telephone
CI: confidence interval
CUA: cost‐utility analysis
ECEC: early childhood education and care
EPOCH: early prevention of obesity in childhood
FAB: food, activity, and breastfeeding
FDC: family day care
FTE: full‐time equivalent
HALY: health‐adjusted life year
ICER: incremental cost‐effectiveness ratio
ICT: information and communications technology
InFANT: infant feeding activity and nutrition randomized controlled trial
kg: kilogram
KGFYL: kids‐go for your life!
km: kilometres
LDC: long day care
LGA: local government area
LSAC: longitudinal study of Australian children
m^2^
: metres squared
NZD: New Zealand dollar
POI: prevention of overweight in infancy randomized controlled trial
QALY: quality‐adjusted life year
R&C: Romp & Chomp
S4M: smiles 4 miles
USD: United States dollar
WHO: World Health Organization

## 1 INTRODUCTION

Early childhood obesity is a significant public health issue, with approximately 40 million (5.9%) children aged under five classified as having overweight or obesity worldwide.^1^ Childhood obesity negatively affects psychosocial, respiratory, orthopaedic, endocrine and reproductive health.^2^ The condition is also associated with economic consequences, including increased healthcare costs and indirect costs such as school performance or lost productivity.^3^ Evidence also suggests that children and adolescents with overweight and obesity are at increased risk of overweight in adulthood.^2^, ^3^

The prevention of overweight and obesity in the early years of life (i.e., in the first 5 years of life) is of increasing importance internationally, with recognition that the behavioural and biological responses of a child to obesogenic environments can be shaped from a young age.^1^ The World Health Organization (WHO) Commission on Ending Childhood Obesity^1^ outlined a comprehensive package of recommendations to address childhood obesity, including multi‐sectoral, multi‐faceted approaches to prevention. As part of this comprehensive approach, community‐wide interventions, involving community capacity building and engagement with population‐level obesity prevention strategies, have been recognized as promising in achieving modest reductions in population weight gain among children.^4^, ^5^ Evidence also suggests that community‐wide interventions may represent an equitable approach to obesity prevention intervention, which may be of particular importance considering the socioeconomic patterning of obesity starts from a very young age.^6^

Given scarce societal resources, it is important that interventions to reduce childhood obesity represent good value for money. To have an impact at the population level, effective and cost‐effective obesity prevention interventions need to be scaled‐up and widely available.^7^ Limited evidence currently exists on the cost‐effectiveness of interventions aiming to prevent overweight and obesity in the preschool‐age population.^8^ Published literature primarily focuses on school‐based strategies and the impacts of community‐wide interventions on school‐age children.^4^, ^5^, ^9^ In addition, much of the published economic evaluation literature has modelled the costs and effects of community‐wide interventions in primary‐school aged children over their lifetime, to estimate the longer‐term health benefits and healthcare cost‐savings from the prevention of chronic diseases into adulthood (i.e.,^5^, ^10^). No published studies have estimated the cost‐effectiveness of community‐wide interventions in early childhood populations across shorter time horizons, accounting for the health benefits and healthcare cost‐savings that might accrue throughout childhood and adolescence.^11^ Yet such evidence is important because it could assist decision making in a shorter‐term, policy‐relevant timeframe.^2^

This paper aims to estimate the cost‐effectiveness of a community‐wide early childhood obesity prevention intervention—the Romp & Chomp (R&C) intervention—assuming it was hypothetically scaled‐up and nationally delivered to all Australian children from 0 to 5 years of age. R&C was a quasi‐experimental trial of a multi‐setting, multi‐strategy, community‐wide obesity prevention intervention conducted in Geelong, Victoria, Australia from 2004 to 2008 that targeted children aged from 0 to 5 years through community capacity building (i.e., professional training, support provision to early childhood environments to favour obesity prevention) and environmental changes in early childhood education and care (ECEC) settings.^12^ These ECEC settings comprised long day care centres, family day care services, and preschools.^12^ The intervention was designed in collaboration with key stakeholders, including the regional health service, local government and state government departments of health and education. Intervention strategies involved health promotion and activities designed to develop sustainable policy, and sociocultural and environmental changes in early childhood settings. R&C demonstrated a significant difference in body mass index (BMI) (−0.06 kg/m^2^ [95% CI: −0.10; −0.01, *p* <0.01]) among 3.5‐year‐olds between the intervention and comparator groups,^12^ however no economic evaluation was undertaken at the time of the trial.

We undertook modelled cost‐effectiveness analysis (CEA) and cost‐utility analysis (CUA) over a 10‐year time horizon, from 5 to 15 years of age. The research question was: From a funder perspective, would the R&C intervention delivered nationally to the population of all Australian children aged from 0 to 5 years be cost‐effective by age 15 years (in terms of cost per BMI unit avoided at age 15 years and cost per quality‐adjusted life year (QALY) gained by age 15 years), compared to a “no intervention” comparator?

## 2 METHODS

Modelled CEA and CUA were undertaken, using published estimates of effectiveness of the R&C intervention^12^ applied to a nationally representative cohort and using intervention cost estimates from a detailed retrospective costing analysis. CEA compares the cost of an intervention relative to control with the intervention outcome measured in natural units (i.e., cost per BMI unit avoided). CUA compares the cost of an intervention relative to control with the intervention outcome measured in a metric incorporating the impact on both quality and quantity of life (i.e., cost per QALY gained).

To conduct our modelled economic evaluation, we extrapolated the within‐trial intervention costs and effects to a nationally representative cohort of the Australian children aged 0 to 5 years. A previously published health economic model, the Early Prevention of Obesity in CHildhood (EPOCH) model,^11^ was used to estimate the health benefits, healthcare cost‐savings and incremental cost‐effectiveness of the R&C intervention beyond the duration of the R&C intervention efficacy trial. The economic evaluation followed the recommendations of the Second Panel on Cost‐Effectiveness in Health and Medicine.^13^ Analyses were reported following the Consolidated Health Economics Evaluation Reporting Standards^14^ (Appendix S1, Supporting Information).

### 2.1 The intervention and comparator

R&C was a community‐wide obesity prevention intervention that targeted children aged 0 to 5 years across the City of Greater Geelong and Borough of Queenscliffe in Victoria, Australia (*n* ~ 12 000), their families and the organizational caregivers at ECEC settings.^12^ The intervention emphasized community capacity building and sustainable changes in policy, sociocultural and physical environments in ECEC using a socioecological framework to encourage healthy eating, active play, reduced screen time and attainment of healthy weight.^12^ Through professional training, policy development and messaging materials, the intervention focused on four key messages: (1) daily active play, (2) daily water and fewer sweet drinks, (3) daily fruit and vegetables and (4) less screen time.^12^

During the intervention phase of R&C, there were two other health promotion programs available in ECEC in the Geelong and Queenscliffe local government areas (LGAs): Smiles 4 Miles (S4M) and Kids‐Go for your Life! (KGFYL),^12^ although the extent of implementation in each setting and factors such as fidelity are unknown. S4M was a program initiated by Dental Health Services Victoria to improve the oral health of at‐risk children and their families across Victoria by emphasizing five key messages: (1) drink well, (2) eat well, (3) clean well, (4) stay well and (5) play well.^15^ KGFYL was a state‐wide program which aimed to increase healthy eating and physical activity of Victorian children through the promotion of six key messages: (1) limit food, (2) move, play and go, (3) turn off, switch to play, (4) tap into water every day, (5) stride and ride and (6) plant fruit and vegetables in your lunchbox.^16^ Given the obvious synergies, these programs were delivered with the R&C intervention as an integrated intervention package in the intervention region.^12^ Further details of the R&C, S4M and KGFYL intervention have been reported elsewhere.^12^, ^15^, ^16^

We defined the comparator for both the CEA and the CUA as a nationally representative cohort of Australian children that did not receive the R&C intervention. Given the lack of available data on implementation of S4M and KGFYL we assumed that the comparison group received no intervention (i.e., this group did not receive R&C, S4M or KGFYL interventions). This may result in an overestimation of intervention cost as compared to the cost of the control, but was deemed to be the most conservative approach to estimating cost‐effectiveness.

### 2.2 Measurement of effectiveness

Intervention effect was estimated using a repeat cross‐sectional quasi‐experimental design to measure the differences in outcomes between the population exposed to the R&C intervention (the intervention sample) compared to the comparison population drawn from other LGAs across Victoria.^12^ Height and weight data were collected by trained Maternal Child Health nurses as part of routine Key Ages and Stages health checks.^12^ The survey collected data pre‐ and post‐ intervention in the intervention and non‐intervention LGAs; therefore, the intervention effect sizes were the average of all the children in the intervention communities compared to those of the non‐intervention communities.^12^ Data comprised children who had attended their 2‐ and 3.5‐year‐old health checks in 2004 and 2007.^12^ In the 3.5‐year‐old intervention sample, the R&C intervention demonstrated a statistically significant reduction in BMI of −0.06 kg/m^2^ (95% CI: −0.10; −0.01, *p* <0.01) relative to the control group.^12^ Our base case analysis assumed effect maintenance until age 15 years, meaning that the children remained in and moved on a BMI trajectory informed by the EPOCH model^17^ based on national data^18^ and according to their BMI at age 3.5 years.

### 2.3 Target population and setting

To conduct our economic evaluation, we assumed that the R&C intervention was scaled up and delivered nationally to all Australian children aged from 0 to 5 years (*n* = 1 906 075)^19^ given the evaluation sample was a cross‐section of all children in the intervention age group using Maternal and Child Health Key Age and Stage health checks.^12^ The intervention settings for the national modelling included all ECEC settings (*n* = 12 463^20^, ^21^): family day care (FDC) (*n* = 906^20^, ^21^), centre‐based long day care (LDC) and preschools (*n* = 11 557^20^). FDC is a type of formal care provided by a registered early childhood educator and carer in a home setting for a small group of children.^22^ Centre‐based LDC is delivered by trained educators and carers, and may involve an integrated preschool program.^22^ Preschool programs (called “Kindergartens” in Victoria) are noncompulsory, government‐funded learning programs, normally delivered by early childhood educators to children within 1 or 2 years before starting formal education (typically, when children are aged between 3 and 5 years).^20^

### 2.4 Resource use and costs

Intervention costs from a funder perspective were estimated retrospectively, using trial records and micro‐costing techniques. All assumptions on how the intervention would be implemented at scale were based on the existing literature on community‐wide obesity prevention intervention,^23^ the management structure reported in trial records (unpublished documents) and in consultation with members of the R&C research team.

Costs were categorized as: time costs, travel costs and intervention material costs. Time costs were estimated using published wage rates including salary on‐costs (i.e., overhead costs, superannuation, employer taxes, compensation, and leave loading).^24^ We assumed that an average of one state‐level full‐time equivalent (FTE) Project Administrator would be required in each Australian state and territory (*n* = 8), to develop policy, manage the intervention implementation within each jurisdiction and provide online training to an average of one 0.5FTE Health Promotion Officer located within each Australian LGA (*n* = 562^25^). While some smaller LGAs may share a Project Administrator and Health Promotion Officer fraction, some larger LGAs may need more capacity. The Health Promotion Officer would, consistent with the R&C implementation, assist the localized intervention implementation within each LGA, train early childhood educators and carers, develop and implement policies at local ECEC settings to engage participants in the messages of R&C. The time cost of 1FTE Web Support Technician was required to maintain, update an intervention website, and to ensure the ongoing availability of online intervention materials.^24^, ^26^ Time costs for early childhood carers from FDC (*n* = 906^20^, ^21^) and educators from LDC and preschools (*n* = 11 557^20^) were estimated as 1 h per educator or carer per year for training in R&C key messages, and 0.5 h per year for the time cost of presenting a sweet drink demonstration to parents. It was assumed that ECEC managers would spend approximately 16 h each year aligning policies with the intervention messages and monitoring the intervention implementation at each setting (*n* = 12 463^20^, ^21^). Time costs for dentists to engage with parents and preschool staff training were assumed to be 1 h per year for each ECEC setting (*n* = 12 463^20^, ^21^).

Costs would be incurred by each LGA‐level Health Promotion Officer (*n* = 562^25^) for travel to local events to promote intervention messages, and to ECEC settings to provide intervention training to early childhood educators and carers. Travel costs were estimated using published guidelines.^27^ The travel distance for each one‐way trip was assumed to be 16 km, which was based on the average commute distance of Australian residents.^28^ This assumption was tested in sensitivity analyses (see Section 2.6).

Intervention material costs consisted of marketing and promotional materials provided to ECEC settings and participants. Each ECEC setting received a program print, early childhood services toolkit documents, display posters, fact sheets, and stickers to encourage healthy eating and active play.^12^ An annual sweet drink demonstration was presented to parents by early childhood educators.^12^ While the base case analysis assumed that participants received most intervention materials electronically (i.e., newsletters, information postcards, tip sheets), intervention participants received a water bottle and lunch bag.^12^, ^15^, ^16^ We assumed the intervention was promoted at four local events per LGA per year (*n* = 2 248 presences nationally), based on trial records (unpublished documents). This incurred a stall fee and promotional materials (i.e., a trestle table, show bags, lunch box brochures). Unit costs for intervention materials and the cost of acquiring and annually maintaining the intervention website domain and hosting were estimated using market prices (Table S3).

The intervention was costed assuming it was in steady state, running at its full effectiveness potential (i.e., excluding costs associated with research and intervention planning and development). In the base case analysis, intervention costs were assumed to be borne by all children aged 0 to 5 years. All costs were estimated in 2018 Australian dollars (AUD1 = USD0.70^29^), and if required, unit costs were adjusted to 2018 values using the Consumer Price Index.^30^ All future costs and benefits were discounted at 5% annually.^31^ Intervention costs by major cost category and a detailed summary of intervention costs are presented in Tables 1 and S3.

**TABLE 1:** Romp & Chomp intervention cost categories, assumptions and data sources

Parameters	Assumption	Data source
Time cost		
Project administrators	1 Project Administrator (1 FTE) per Australian state/territory (*n* = 8)	“Contract, Program and Project Administrators” fulltime weekly salary,^24^, ^26^, ^32^ including 14.5% on‐costs and 17.5% leave loading^26^
Health promotion officers	1 Health Promotion Officer (0.5 FTE) per LGA (*n* = 562)^25^	“Other Health Diagnostic and Promotion Professionals” fulltime weekly salary,^24^, ^26^, ^32^ including 14.5% on‐costs and 17.5% leave loading^26^
Web support technician	1 Web Support Technician (1FTE)	“ICT Support Technicians” fulltime weekly salary,^24^, ^26^, ^32^ including 14.5% on‐costs and 17.5% leave loading^26^
Early childhood carers	1 h training and 0.5 h of sweet drink demonstration, 1 Child Carer per FDC (*n* = 906)^20^, ^21^	“Child Carers” hourly rate,^24^, ^26^ including 14.5% on‐costs and 17.5% leave loading^26^
Early childhood educators	1 h training and 0.5 h of sweet drink demonstration, 1 Child Carer per LDC and preschool (*n* = 11 557)^20^	“School Teachers” hourly rate,^24^, ^26^ including 14.5% on‐costs and 17.5% leave loading^26^
ECEC managers	16 h aligning ECEC settings policies to intervention messages (*n* = 12 463)^20^, ^21^	“Education, health and welfare services managers” hourly rate,^24^, ^26^ including 14.5% on‐costs and 17.5% leave loading^26^
Dentist	1 h engaging with parents and early childhood carers and educators (*n* = 12 463)^20^, ^21^	“Health therapy professional” hourly rate,^24^, ^26^ including 14.5% on‐costs and 17.5% leave loading^26^
Travel cost		
Health promotion officers travel to attend festivals	4 festival presences within each LGA per year (*n* = 2 248), 16 km each way to festival locations^28^	Car expenses, Australian Taxation Office^27^
Health promotion officers travel to provide training session for FDC, LDC and preschools	16 km each way trip to FDC, LDC and preschool^28^	
Material and equipment cost		
Festival stall booking	(*n* = 2 248)	Market rates (Table S3)
Resources for festival attendances	Resources required for each festival presence: 1 A2 trestle table 200 balloons 200 show bags 200 crayon packages 200 lunch box brochures	
Printed training booklets for Health Promotion Officers and ECEC settings	1 training booklet per Health Promotion Officer (*n* = 562)^25^ and ECEC setting (*n* = 12 463)^20^, ^21^	Market rates (Table S3)
Marketing and promotional materials for ECEC	4 units of each marketing and promotion material type for each ECEC setting (*n* = 12 463)^20^, ^21^	
Resources for “sweet drinks demonstrations” (i.e., sugar, carbonated drink, topping, coffee)	1 30‐min sweet drink demonstration delivered at each early education and care setting each year	
KGFYL drink bottles	1 drink bottle for each participant (*n* = 884 179)^19^	
S4M lunch boxes	1 lunch box for each participant (*n* = 884 179)^19^	
Web domain	All intervention materials for participants are online	
Web hosting	All intervention materials for participants are online	

Abbreviations: ECEC, early childhood education and care; FDC, family day care; FTE, full‐time equivalent; ICT, Information and communications technology; KGFYL, Kids‐Go for your life; LDC, long day care; R&C, Romp & Chomp; LGA, local government area; S4M, Smiles 4 Miles.

### 2.5 Modelling method

A deterministic micro‐simulation model (the EPOCH model)^17^ was used to predict individual level child BMI trajectories, weight status and associated QALYs and healthcare costs from age 4 to 15 years, extrapolating the trial‐based intervention effects nationally to children in the target age group. Simulated BMI and QALYs to age 15 years were modelled using trial data at age 3.5 years. Each child was set on a different BMI trajectory based on the different starting BMI measured at the end of the trial.^17^, ^33^ Modelled CEA estimated the incremental cost per BMI unit avoided (AUD/BMI unit avoided) at age 15 years and modelled CUA estimated the incremental cost per QALY gained (AUD/QALY gained) to age 15 years, compared to a no‐intervention comparator.

Data from the LSAC used as the representative national level input population for our modelled economic evaluation.^18^ The LSAC is a national, comprehensive, and multi‐disciplinary Australian dataset of children from two cohorts, the “baby” (“B”) cohort and the “kindergarten” (“K”) cohort, followed from aged 0–1 to 4–5 years, respectively.^18^ The B cohort was selected as our input population for our analyses given the data collection of the B cohort corresponds to the roll out of R&C. Intervention effect size (−0.06 kg/m^2^) was applied to child BMI at age 4/5 years to estimate BMI trajectories of the intervention group to age 14/15 years and compared to the trajectories of the same cohort without any intervention effects applied. QALY weights associated with child weight status to inform the estimation of QALYs were obtained from a recent systematic review and meta‐analysis.^34^ The QALY weights of children with healthy weight, overweight and obesity were 0.85, 0.83 and 0.82, respectively.^34^ The classification of weight status was based on WHO growth standards.^35^ Healthcare costs of participants to age 15 years were modelled following a ‘top down’ method, using administrative records of annual hospital,^36^ doctor and medical costs by age^37^ adjusted by weight status.^38^ All analyses were conducted in Stata version 16.1.^39^ A more detailed summary is provided in Supporting Information S4.

We estimated the joint uncertainty around costs and QALYs by creating 1 000 bootstrapped samples, which were then used to calculate the probability of the intervention being cost‐effective compared to the comparator at different willingness to pay thresholds.^33^ The bootstrapping accounts for individual level heterogeneity in simulated costs, BMI and QALYs, whilst uncertainty in input assumptions was investigated through sensitivity analysis. Results were presented as incremental cost‐effectiveness ratios (ICERs), defined as the incremental cost of implementing the intervention divided by the incremental effectiveness. The commonly adopted cost‐effectiveness threshold for CUA of AUD50 000/QALY gained^40^ was used to determine cost‐effectiveness. Modelled results were plotted on a cost‐effectiveness plane, which is a visual representation of incremental costs and incremental effects corresponding to the 1 000 bootstrapped iterations. When the point estimates fall in the north‐east quadrant of the cost‐effectiveness plane, the intervention is more costly and more effective. Cost‐effectiveness acceptability curves were also generated, depicting the probability of the intervention being cost‐effective for a range of different willingness‐to‐pay thresholds.

### 2.6 Sensitivity analysis

A series of univariate and multivariate sensitivity analyses were performed to evaluate the impact of assumptions made (Table 2). In sensitivity analysis 1, intervention costs were varied, assuming that they were borne only by the population in which the intervention effect was modelled (i.e., children aged 4–5 years, n = 642 178^19^). In sensitivity analysis 2, higher intervention costs were assumed, based on: (i) intervention resources for participants being paper‐based; (ii) the Health Promotion Officer at 562 LGAs being employed at 1 FTE; (iii) the allocation of 2 h each year for early childhood carers and educators to attend training and perform the sweet drink demonstration; (iv) the allocation of 1.5 h each year for dentists to engage with parents and staff training and (v) a longer distance of 31.2 km was travelled by Health Promotion Officers within LGAs to deliver training and to attend festivals.^41^ In sensitivity analysis 3, a “worst case” scenario was also examined, using the low confidence interval (CI) of the intervention effect on BMI (i.e., −0.01 kg/m^2^)^12^ and the higher intervention cost. In sensitivity analysis 4, the discount rate was reduced from 5% to 3%.^13^

**TABLE 2:** Summary of Romp & Chomp intervention base case and sensitivity analyses

	Base case	Sensitivity analysis 1: Intervention costs borne only by children aged 4 to 5 years	Sensitivity analysis 2: High intervention cost estimate	Sensitivity analysis 3: Worst case^a^	Sensitivity analysis 4: 3% discount rate
Number of children intervention costs borne by	1 906 075^19^	642 178^19^	1 906 075^19^	642 178^19^	1 906 075^19^
BMI effect size at age 3.5 years	−0.06 kg/m^2^			−0.01 kg/m^2^	−0.06 kg/m^2^
Intervention costs assumptions	As per Table 1		As per Table 1, except: Health Promotion Officers (*n* = 652) employed at 1 FTE. 2 h allocated to training and sweet drink demonstration by Child Carers and Preschool Teachers. 1.5 h allocated for dentists to engage with parents and early childhood carers and educators training. Intervention materials for participants were paper based. Travel distance to ECEC settings and festivals of 31.2km^41^		As per Table 1
Discount rate	5%				3%

Abbreviations: BMI, body mass index; ECEC, early childhood education and care; FTE, full‐time equivalent; kg, kilogram; km, kilometre; m, metre.

^a^ Lower CI of intervention effect, high‐cost estimate and intervention cost only borne by children aged 4–5 years.

## 3 RESULTS

Under the base case assumptions, the intervention costs totalled AUD177 536 705 per year, with the annual cost per participant averaging AUD93 (Table 3). By age 15 years, the modelled healthcare cost‐saving was AUD15 per participant as compared to the no‐intervention comparator (Table 3).

**TABLE 3:** Cost‐effectiveness results of the scaled‐up Romp & Chomp intervention, modelled from age 4 to 15 years

	Base case	Sensitivity analysis 1: Intervention costs borne only by children aged 4 to 5 years	Sensitivity analysis 2: High intervention cost estimate	Sensitivity analysis 3: Worst case^a^	Sensitivity analysis 4: 3% discount rate
Total intervention cost per year (2018 AUD)	$177 536 705		$304 902 407		$180 836 160
Mean intervention cost per participant (2018 AUD)	$93	$276	$160	$475	$95
Mean healthcare cost saving per participant (2018 AUD)	$15			$2	$17
Incremental total cost (2018 AUD) (95% CI)	$78 ($54; $103)	$261 ($234; $286)	$145 ($121; $170)	$472 ($450; $497)	$78 ($48; $109)
CEA results at aged 15 years					
Mean BMI unit avoided (95% CI)	0.07 (−0.01; 0.16)			0.01 (−0.07; 0.08)	0.09 (−0.00; 0.19)
Mean ICER, AUD per BMI unit avoided (95% CI)	$1 126 (Dominated^b^; $5 958)	$3 767 (Dominated^b^; $17 683)	$ 2 089 (Dominated^b^; $9 939)	$40 719 (Dominated^b^; $173 331)	$871 (Dominated^b^; $6 035)
CUA results at aged 15 years					
Mean QALY gained (95% CI)	0.003 (−0.006; 0.012)			0.0005 (−0.008; 0.009)	0.003 (−0.008; 0.015)
Mean ICER, AUD per QALY gained (95% CI)^b^	$26 399 (Dominated^b^; $246 826)	$88 332 (Dominated^b^; $875 591)	$48 974 (Dominated^b^; $481 265)	$956 146 (Dominated^b^; $1 373 912)	$22 894 (Dominated^b^; $182 830)
Probability of being cost‐effective	64%	31%	53%	1.6%	64%
Overall result	Cost‐effective	Not cost‐effective	Cost‐effective	Not cost‐effective	Cost‐effective

Abbreviations: AUD, Australian dollars; BMI, body mass index; CEA, cost‐effectiveness analysis; CI, bootstrapped confidence interval; CUA, cost‐utility analysis; ICER, Incremental cost‐effectiveness ratio; QALY, quality‐adjusted life year.

^a^ Lower CI of intervention effect, high‐cost estimate and intervention cost only borne by children aged 4–5 years.

^b^ Dominated: the intervention results in higher costs and lower health benefits.

**FIGURE 1:**
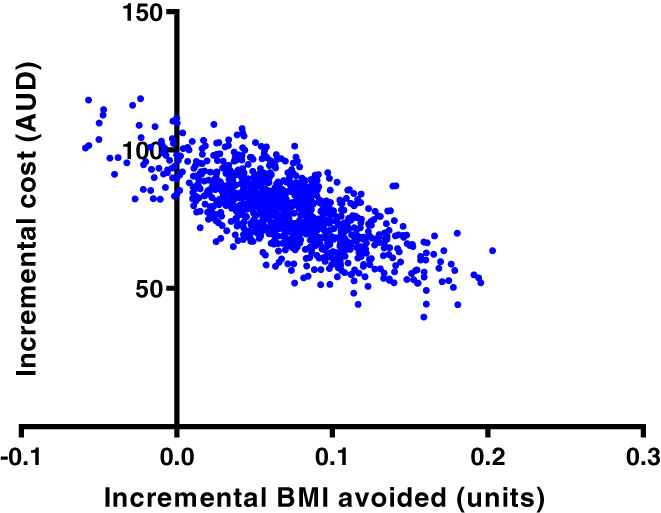
Cost‐effectiveness plane at age 15 years—base case cost‐effectiveness analysis. AUD, Australian dollar; BMI, body mass index

The intervention led to a 0.07 reduction in BMI units at age 15 years compared to the no intervention comparator, resulting in an ICER of AUD1 126 per BMI unit avoided at age 15 years (Table 3 and Figure 1).

**FIGURE 2:**
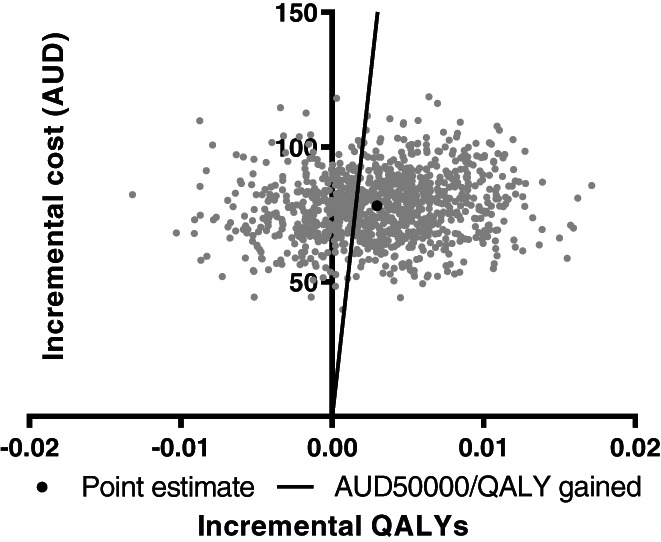
Cost‐effectiveness plane at age 15 years—base case cost‐utility analysis. AUD, Australian dollar; QALYs, quality‐adjusted life years

At age 15 years, 0.003 QALYs were gained per child, as compared to the no intervention comparator (Table 3). The national delivery of the R&C intervention, with mean ICER of AUD26 399/QALY gained, has a 64% probability of being cost‐effective compared to the commonly accepted AUD50 000/QALY gained threshold (Figure 2).

The intervention is not cost‐effective when intervention costs are borne only by children aged 4 to 5 years (sensitivity analysis 1), as a result of the higher intervention cost per participant (AUD276; approximately three times higher than the base case cost). The intervention is also not cost‐effective under worst case assumptions (sensitivity analysis 3), as a result of the intervention effect being six times lower and the mean intervention cost per participant more than five times higher than in the base case analysis (Table 3).

## 4 DISCUSSION

Our analysis provides important evidence of the likely cost‐effectiveness of a scaled‐up community‐wide obesity prevention intervention for children from 0 to 5 years. The results indicate a 64% probability of R&C being cost‐effective under base case assumptions, which include web‐based intervention components and low personnel time costs. Since the R&C trial in 2004, obesity prevention interventions with intervention components delivered via technology have become increasingly popular^42^, ^43^. Therefore, our base case assumptions included the adoption of electronic intervention resources. This provides a low‐cost and flexible mode of intervention delivery, particularly given the heterogeneity in ECEC settings between Australian states and territories and the constantly evolving nature of the ECEC environment. Utilizing technology to facilitate the implementation of the intervention may also streamline personnel time required to oversee the program from Project Administrators and Health Promotion Officers.

The key inputs which influence the cost‐effectiveness results of the R&C intervention include the population size that bears the intervention cost, and the intervention effect estimate (sensitivity analysis 1, 3). Compared to other childhood obesity prevention interventions, the R&C effectiveness estimates were not substantial (Appendix S7). For example, the R&C effectiveness estimate of −0.06 kg/m^2^ was lower than that of other early childhood obesity prevention interventions, including the Prevention of Overweight in Infancy (POI) (−0.26 kg/m^2^),^33^ Be Active Eat Well (−0.28 kg/m^2^),^10^ Project Energize (−0.50 kg/m^2^ in children aged 6–8 years or − 0.55 kg/m^2^ in children aged 9–11 years).^44^ It should however be noted that plausible effect sizes for population level interventions may be relatively small, and are dependent on a number of factors including the characteristics of the intervention and the target population. The R&C intervention was associated with a reduction in the prevalence of overweight/obesity that was five times more than in the comparison sample of 3.5 year old children,^12^ with significant potential for population level health benefits should the intervention be delivered at scale. This highlights the need for an efficient monitoring system in place to evaluate the ongoing intervention effect on participants, and to ensure the intervention is effective within the willingness‐to‐pay threshold if implemented nationally and over time. For instance, it is likely that the intervention material should be redesigned periodically to reinforce the intervention message, to adhere to the intervention message, to updated nutrition guidelines or to reflect evolving changes in the ECEC setting. Future research should investigate this matter.

While the healthcare cost savings arising from the R&C intervention were not large (i.e., an incremental saving of only AUD15 per participant in the base case analysis), our evaluation was able to capture the mid‐term (within the first 15 years of life) quality of life impacts. Given that evidence suggests that the detrimental health impacts of unhealthy BMI in childhood are likely to carry into adulthood and increase susceptibility to various chronic non‐communicable diseases,^3^ the relatively small impacts of the intervention on participant weight and healthcare cost‐savings at age 15 years may potentially have more significant longer‐term health and healthcare cost‐saving implications which we have not estimated here. Other potential benefits, related to productivity or child development outcomes^11^ are also not included in our analyses.

Five Australasian early childhood obesity prevention interventions in children aged from 0 to 2 years—Healthy Beginnings,^45^ Communicating Healthy Beginnings Advice by Telephone (CHAT),^46^ Infant Feeding Activity and Nutrition trial (InFANT),^47^ Prevention of Overweight in Infancy (POI)^33^ and NOURISH^48^—were recently costed used similar costing methodologies to those employed here.^49^ The R&C intervention had a lower cost per participant compared to four of the five early childhood obesity prevention interventions, except the CHAT‐SMS arm,^46^ despite the addition of extra costs related to the hypothetical implementation at scale for this analysis. It should be noted however that the five early childhood obesity prevention interventions all included more resource intensive intervention components delivered to a younger target population (i.e., from 0 to age 2 years) than R&C.^49^ The economies of scale of the hypothetical national implementation of R&C also resulted in some cost efficiencies as compared to these studies.^49^

A full economic evaluation of the POI intervention has also recently been published, using the EPOCH model to estimate the cost‐effectiveness of sleep, nutrition, and physical activity intervention targeting children during the first 2 years of life.^33^ Modelled results for the POI‐Sleep intervention arm demonstrated a cost‐effective ICER of AUD18 125/QALY gained at age 15 years.^33^ While R&C has lower intervention effect on BMI (−0.06 kg/m^2^) at age 15 years, it was also less costly given the less intensive mode of delivery.^33^

Although it is difficult to directly compare cost‐effectiveness results of R&C with studies using different economic evaluation methodologies, conducted in different populations and evaluated over a different time horizon, Appendix S7 provides some results from published economic evaluations of other community‐wide interventions. In Australia, a cost‐effectiveness study, modelling a hypothetical scale‐up of a 3‐year community‐led obesity prevention intervention targeting children aged 5 to 18 years attending government‐run primary and secondary schools across Australia, reported a total intervention cost of AUD878M in 2010 (≈AUD1 036M [2018 value]^30^) with cost per child of AUD499 (≈AUD589 [2018 value]^30^).^5^ The intervention was cost‐effective when modelled over the lifetime, with an ICER of AUD8 155 (≈AUD9 619 [2018 value]^30^) per health‐adjusted life year gained.^5^

An analysis of the Be Active Eat Well (BAEW) intervention in Australia also demonstrated cost‐effectiveness.^10^ The total modelled intervention cost per child for BAEW was much higher than the cost for R&C, at AUD344 (2006 value; AUD453 2018 value^30^) given the more substantial expenditure allocated for personnel costs and venue hire costs.^10^ The cost per BMI unit avoided of BAEW was however relatively similar to R&C (AUD399 (≈AUD525 [2018 value]^30^ versus AUD500),^10^ due to the larger BAEW effect sizes (−0.28 kg/m^2^ versus −0.06 kg/m^2^ change in BMI for R&C) and the longer modelling time horizon.^10^

When compared with a community‐wide intervention in New Zealand, the R&C cost per participant is relatively high compared with that of Project Energize (NZD44.96 (2010 value) (≈AUD45.72 (2010 value)^29^ ≈ AUD54 (2018 value)^30^ versus AUD93).^44^ The ICERs of Project Energize (NZD30 438/QALY (≈AUD30 580 (2010 value) ≈ AUD27 171 (2018 value)^29^, ^30^) for children aged 6 to 8 years and NZD24 690/QALY (≈AUD24 806 (2010 value) ≈ AUD29 260 (2018 value)^29^, ^30^) for children aged 9 to 11 years) are comparable to that of R&C.^44^ This is likely due to Project Energize's modelling including an intervention effect decay of 1% annually after the 5‐year intervention duration.^44^ This highlights the need to further investigate the impact of intervention effect sustainability on cost‐effectiveness results in future research.

Finally, A Pilot Program for Lifestyle and Exercise (APPLE) targeted primary school children and aimed to change the environment of schools and the wider community utilizing community activity coordinators.^50^ The cost per participant of APPLE is much higher than the cost of the R&C intervention (NZD1 281 over 2 years (2006 value) ≈ AUD1 214 (2006 value)^29^ ≈ AUD1 591 (2018 value)^30^)^50^ given the intervention was more resource‐intensive.

Our cost‐effectiveness results suggest that R&C should be considered as part of a package of interventions to reduce the prevalence of obesity in children. The key aims and messages of R&C are well‐aligned with Australian state and national strategies to improve important health outcomes for children aged under 5 years,^51^ and given the simple messaging and execution of the intervention it is likely that it will appeal to potential funders and those in ECEC settings. The strengths of this evaluation include the use of intervention effect estimates from a complete dataset of Maternal and Child Health Key Age and Stage health checks^12^ and the robustness of the EPOCH economic model.^17^ The evaluation also goes beyond the economic evaluation of the trial to model the cost‐effectiveness of the intervention should it be implemented nationally. This method provides important economic evidence for policy makers by hypothetically evaluating the intervention in its potential steady nationwide implementation state.

Limitations include the assumptions required to extrapolate costs and effects nationally, although our analysis followed methodologies in the published literature^8^ and we conducted extensive sensitivity analyses. The assumption that some R&C intervention content would be delivered electronically differs from the original intervention design and it is unknown whether these changes in delivery method might affect the intervention effects. Future research should examine the impacts of different delivery modalities. Second, there was no sensitivity analysis conducted to assess how intervention effect decay influences the ICERs although the sustainability of intervention effects post‐intervention were reported to affect the cost‐effectiveness of Project Energize and the BAEW program.^44^, ^50^ Third, probabilistic uncertainty analysis of the range and distribution of intervention effect size and intervention cost per participant was not conducted; instead multiple one‐way sensitivity analyses were carried out to test the impact of changes in input assumptions pertaining to costs and effects. Finally, the long‐term health burden associated with high BMI was not estimated and this is an area for future study.

## 5 CONCLUSIONS

Excess BMI affects Australian children from a very young age, which negatively impacts their quality of life in the short term and causes increased risks of developing chronic diseases in the long term.^3^ R&C was a community‐wide obesity prevention intervention that effectively reduced weight and BMI measures in children aged under 5 years. The economic evaluation of the scale‐up of this intervention suggests that it has a fair probability of being cost‐effective under various sensitivity analyses. The evidence from this evaluation expands the current limited evidence pool of cost‐effective obesity prevention interventions in early childhood and adolescence.

## AUTHOR CONTRIBUTIONS

Huong Ngoc Quynh Tran costed the intervention, with input from Melanie Nichols and Vicki Brown. Alison Hayes, Anagha Killedar and Eng Joo Tan conducted the economic modelling. Huong Ngoc Quynh Tran wrote the first draft of the paper. All authors reviewed and commented on the paper.

## CONFLICT OF INTEREST

The authors declare that the research was conducted in the absence of any commercial or financial relationships that could be construed as a potential conflict of interest.

## Supporting information


**APPENDIX S1**: Supporting Information
