# Supplementary material for: Cost‐effectiveness of scaling up a whole‐of‐community intervention: The Romp & Chomp early childhood obesity prevention intervention
Source: Pediatr Obes. 2022 Mar 17;17(9):e12915. doi: 10.1111/ijpo.12915 (PMC9540361; doi:10.1111/ijpo.12915)
Supplement: Supplementary file 1 — APPENDIX S1: Supporting Information [file IJPO-17-e12915-s001.docx]

**Cost-effectiveness of scaling up a whole of community intervention: the Romp & Chomp early childhood obesity prevention intervention**

Huong Ngoc Quynh Tran^1,2^, Anagha Killedar^3^, Eng Joo Tan^1^, Marj Moodie^1,2^, Alison Hayes^1,3^, Boyd Swinburn^2,4^, Melanie Nichols^2^, Vicki Brown^1,2^

^1^ Deakin Health Economics, Institute for Health Transformation, School of Health and Social Development, Deakin University, Geelong, Victoria, Australia

^2^ Global Obesity Centre (GLOBE), Institute for Health Transformation, School of Health and Social Development, Deakin University, Geelong, Victoria, Australia

^3^ The University of Sydney, Faculty of Medicine and Health, School of Public Health, NSW Australia

^4^ School of Population Health, University of Auckland, Auckland, New Zealand

Corresponding author:

Huong Ngoc Quynh Tran, Deakin Health Economics, Deakin University, Geelong, Victoria, Australia.

Email: [michelle.tran@deakin.edu.au](mailto:michelle.tran@deakin.edu.au)

**Appendix 1 – Consolidated Health Economic Evaluation Reporting Standards (CHEERS) checklist**

| **Section/item** | **Item No** | **Recommendation** | **Reported on page No/line No** |
| --- | --- | --- | --- |
| Title and abstract | | | |
| Title | 1 | Identify the study as an economic evaluation or use more specific terms such as “cost-effectiveness analysis”, and describe the interventions compared. | Page 1, line 1-3 |
| Abstract | 2 | Provide a structured summary of objectives | Page 3 |
| Introduction | | | |
| Background and objectives | 3 | Provide an explicit statement of the broader context for the study. | Page 5-7 |
|  |  | Present the study question and its relevance for health policy or practice decisions. | Page 5-7 |
| Methods | | | |
| Target population and subgroups | 4 | Describe characteristics of the base case population and subgroups analysed, including why they were chosen | Page 9 |
| Setting and location | 5 | State relevant aspects of the system(s) in which the decision(s) need(s) to be made. | Page 9 |
| Study perspective | 6 | Describe the perspective of the study and relate this to the costs being evaluated. | Page 10 |
| Comparators | 7 | Describe the interventions or strategies being compared and state why they were chosen. | Page 8 |
| Time horizon | 8 | State the time horizon(s) over which costs and consequences are being evaluated and say why appropriate. | Page 7 |
| Discount rate | 9 | Report the choice of discount rate(s) used for costs and outcomes and say why appropriate. | Page 12, 16 |
| Choice of health outcomes | 10 | Describe what outcomes were used as the measure(s) of benefit in the evaluation and their relevance for the type of analysis performed. | Page 15-16 |
| Measurement of effectiveness | 11a | Single study-based estimates: Describe fully the design features of the single effectiveness study and why the single study was a sufficient source of clinical effectiveness data. | Page 8-9 |
|  | 11b | Synthesis-based estimates: Describe fully the methods used for identification of included studies and synthesis of clinical effectiveness data. | NA |
| Measurement and valuation of preference based outcomes | 12 | If applicable, describe the population and methods used to elicit preferences for outcomes. | Page 15 |
| Estimating resource use and costs | 13a | Single study-based economic evaluation: Describe approaches used to estimate resource use associated with the alternative interventions. Describe primary or secondary research methods for valuing each resource item in terms of its unit cost. Describe any adjustments made to approximate to opportunity costs. | Page 9-12 |
|  | 13b | Model-based economic evaluation: Describe approaches and data sources used to estimate resource use associated with model health states. Describe primary or secondary research methods for valuing each resource item in terms of its unit cost. Describe any adjustments made to approximate to opportunity costs. | NA |
| Currency, price date and conversion | 14 | Report the dates of the estimated resource quantities and unit costs. Describe methods for adjusting estimated unit costs to the year of reported costs if necessary. Describe methods for converting costs into a common currency base and the exchange rate. | Page 11 |
| Choice of model | 15 | Describe and give reasons for the specific type of decision-analytical model used.  Providing a figure to show model structure is strongly recommended. | Page 15-16 |
| Assumptions | 16 | Describe all structural or other assumptions underpinning the decision-analytical model. | Page 15-16 |
| Analytical methods | 17 | Describe all analytical methods supporting the evaluation. This could include methods for dealing with skewed, missing, or censored data; extrapolation methods; methods for pooling data; approaches to validate or make adjustments (such as half cycle corrections) to a model; and methods for handling population heterogeneity and uncertainty. | Page 15-16 |
| Results | | | |
| Study parameters | 18 | Report the values, ranges, references, and, if used, probability distributions for all parameters. Report reasons or sources for distributions used to represent uncertainty where appropriate. Providing a table to show the input values is strongly recommended. | Page 18 |
| Incremental costs and outcomes | 19 | For each intervention, report mean values for the main categories of estimated costs and outcomes of interest, as well as mean differences between the comparator groups. If applicable, report incremental cost-effectiveness ratios. | Page 18-20 |
| Characterising uncertainty | 20a | Single study-based economic evaluation: Describe the effects of sampling uncertainty for the estimated incremental cost and incremental effectiveness parameters, together with the impact of methodological assumptions (such as discount rate, study perspective). | NA |
|  | 20b | Model-based economic evaluation: Describe the effects on the results of uncertainty for all input parameters, and uncertainty related to the structure of the model and assumptions. | Page 20 |
| Characterising heterogeneity | 21 | If applicable, report differences in costs, outcomes, or cost-effectiveness that can be explained by variations between subgroups of patients with different baseline characteristics or other observed variability in effects that are not reducible by more information. | NA |
| Discussion | | | |
| Study findings, limitations,  generalisability, and current  knowledge | 22 | Summarise key study findings and describe how they support the conclusions reached. Discuss limitations and the generalisability of the findings and how the findings fit with current knowledge. | Page 20-24 |
| Other | | | |
| Source of funding |  | Describe how the study was funded and the role of the funder in the identification, design, conduct, and reporting of the analysis. Describe other nonmonetary sources of support. | Page 26 |
| Conflicts of interest |  | Describe any potential for conflict of interest of study contributors in accordance with journal policy. In the absence of a journal policy, we recommend authors comply with International Committee of Medical Journal Editors recommendations. | Page 25 |

Note: NA: not applicable

**Appendix 2 – Calculation of the number of children aged 0 to 5 years exposed to the intervention in early childhood education and care in Australia**

The number of children aged from 0 to 3 years who were exposed to the intervention was calculated by multiplying the proportion of children in each age group attending formal care ^1^ by the total population of that age group ^2^ in 2017. The number of children aged 4 to 5 years exposed to the intervention was the total number of children aged 4 to 5 years attending preschool program ^3^.

| **Age group** | **Number of children exposed to the intervention** |
| --- | --- |
| **Age 0** | 38,859 |
| **Age 1** | 129,011 |
| **Age 2** | 188,900 |
| **Age 3** | 184,946 |
| **Age 4** | 274,574 |
| **Age 5** | 67,889 |
| **Total** | **884,179** |

**Table S3 – Detailed Romp & Chomp intervention cost summary**

| Parameters | | Mean values | Assumptions | Data source | |
| --- | --- | --- | --- | --- | --- |
| Time cost | | | | |  |
| Project Administrators | | AUD2,180,913 | 1 Project Administrator (1 FTE) per Australian state/territory (n=8) | “Contract, Program and Project Administrators” fulltime weekly salary ^4-6^, including 14.5% on-costs and 17.5% leave loading ^6^ | |
| Health Promotion Officers | | AUD80,356,278 | 1 Health Promotion Officer (0.5 FTE) per LGA (n=562) ^7^ | “Other Health Diagnostic and Promotion Professionals” fulltime weekly salary ^4-6^, including 14.5% on-costs and 17.5% leave loading ^6^ | |
| Web Support Technician | | AUD242,552 | 1 Web Support Technician (1FTE) | “ICT Support Technicians” fulltime weekly salary ^4-6^, including 14.5% on-costs and 17.5% leave loading ^6^ | |
| Early childhood carers | | AUD132,183 | 1 hour training and 0.5 hour of sweet drink demonstration, 1 Child Carer per FDC (n=906) 19,20 | “Child Carers” hourly rate ^5,6^, including 14.5% on-costs and 17.5% leave loading ^6^ | |
| Early childhood educators | | AUD3,200,353 | 1 hour training and 0.5 hour of sweet drink demonstration, 1 Child Carer per LDC and preschool (n=11,557) 19 | “School Teachers” hourly rate ^5,6^, including 14.5% on-costs and 17.5% leave loading ^6^ | |
| ECEC managers | | AUD43,810,800 | 16 hours aligning ECEC settings policies to intervention messages (n=12,463) 19,20 | “Education, health and welfare services managers” hourly rate ^5,6^, including 14.5% on-costs and 17.5% leave loading ^6^ | |
| Dentists | | AUD2,072,646 | 1 hour engaging with parents and early childhood carers and educators (n=12,463) 19,20 | “Health therapy professional” hourly rate ^5,6^, including 14.5% on-costs and 17.5% leave loading ^6^ | |
| Travel cost | | | | |  |
| Health Promotion Officers travel, to attend festivals | | AUD137,018 | Assumed 4 festival attendances within each LGA, 15.98 kilometres each 1-way trip to festival locations ^8^ | Car expenses, Australian Taxation Office ^9^ | |
| Health Promotion Officers travel, to provide training sessions for FDC, LDC and preschool | | AUD759,633 | Assumed 15.98 kilometres each 1-way trip to FDC, LDC and preschool ^8^ | Car expenses, Australian Taxation Office ^9^ | |
| Material and equipment cost | | | | |  |
| Festival stall booking | | AUD1,432,176 | Assumed 4 stall bookings within each LGA (n=2,248) | Market rates ^10-15^ | |
| Resources for festival attendances | | AUD6,785,660 | Resources required for each festival presence:   - 1 A2 trestle - 200 balloons - 200 show bags - 200 crayon packages - 200 lunch box brochures | Market rates ^16-19^ | |
| Printed training booklets for Health Promotion Officers and early education and care settings | | AUD18,575 | Assumed 1 training booklet per Health Promotion Officer (n=562) ^7^ and each early education and care setting (n= 12,463) ^3,20^ | Market rates ^16,21^ | |
| Marketing and promotional materials for early education and care settings | |  |  |  | |
|  | Active play program print | AUD71,095 | Assumed 4 units of each marketing and promotion material type for each early education and care setting (n= 12,463) ^3,20^ | Market rates ^16,18,21^ | |
|  | Active play poster | AUD257,122 |  |  |  |
|  | Daily Fruit & Veg poster | AUD257,122 |  |  |  |
|  | Daily Fruit & Veg fundraising fact sheet | AUD257,122 |  |  |  |
|  | Daily water poster | AUD257,122 |  |  |  |
|  | Fundraising ideas sticker | AUD49,518 |  |  |  |
|  | Energy dense snack display | AUD257,122 |  |  |  |
| Resources for “sweet drinks demonstrations” | | AUD333,470 | Assumed 1 30-minute sweet drink demonstration delivered at each early education and care setting each year. Resources required include sugar, carbonated drink, topping, coffee) | Market rates ^19,22^ | |
| Web domain | | AUD600 | Assumed all intervention materials for participants are online | Market rates ^23^ | |
| Web hosting | | AUD943 | Assumed all intervention materials for participants are online | Market rates ^23^ | |
| *Kids – ‘Go for your life’* | | | | |  |
| Drink bottles for participants | | AUD14,508,454 | Assume 1 drink bottle for each participant each year (n=884,179) ^24^ | Market rates ^18^ | |
| Marketing and promotional materials for early education and care settings | |  |  |  | |
|  | Practical activity sheet - playing alone | AUD17,774 | Assumed 4 units of each marketing and promotion material type for each early education and care setting (n= 12,463) ^3,20^ | Market rates ^16,21^ | |
|  | Practical activity sheet - playing with others | AUD17,774 |  |  |  |
| *Smiles 4 Miles* | | | | |  |
| Marketing and promotion materials for childcare settings | | | | |  |
|  | Oral health newsletters to childcare | AUD73,317 | Assumed 1 unit for each early education and care setting (n= 12,463) ^3,20^ | Market rates ^16,18,21^ | |
|  | Early childhood services toolkit | AUD268,827 |  |  |  |
|  | Drink well fact sheet | AUD257,122 | Assumed 4 units of each marketing and promotion material type for each early education and care setting (n= 12,463) ^3,20^ | Market rates ^16,18,21^ | |
|  | Eat well fact sheet | AUD257,122 |  |  |  |
|  | Clean well fact sheet | AUD257,122 |  |  |  |
|  | Play well fact sheet | AUD257,122 |  |  |  |
|  | Stay well fact sheet | AUD257,122 |  |  |  |
|  | Drink well tip card | AUD257,122 |  |  |  |
|  | Eat well tip card | AUD257,122 |  |  |  |
|  | Clean well tip card | AUD257,122 |  |  |  |
|  | Play well tip card | AUD257,122 |  |  |  |
|  | Stay well tip card | AUD257,122 |  |  |  |
| Lunch boxes for participants | | AUD17,209,316 | Assume 1 lunch box for each participant each year (n=884,179) ^24^ | Market rates ^18^ | |
| Estimated total discounted intervention costs | | **AUD177,536,705** | n=1,906,075 ^24^ |  | |
| Estimated total intervention costs per participant per year if delivered nationally | | **AUD93** |  |  | |

Notes: AUD: Australian dollar; ECEC: early childhood education and care; FDC: family day care; FTE: full-time equivalent; ICT: Information and communications technology; KGFYL: Kids-Go for your life; LDC: long day care; LGA: local government area; S4M: Smiles 4 Miles

**Supporting Information 4 – The EPOCH model**

For a nationally representative sample of 4–5-year-old, we used the EPOCH model ^25^ to model BMI trajectories, utilities and healthcare costs until age 15 years, with and without intervention effects. The sample was taken from the wave 3 measurements of the Birth Cohort of the Longitudinal Study of Australian Children. In wave 3 there were 4311 participants with complete and plausible data on BMI which could be used for the modelling.

The Romp and Chomp effect size was applied by subtracting 0.06 kg/m^2^ (or 0.01 kg/m^2^ for sensitivity analysis 3) from the measured BMI of each participant. Using the EPOCH model, which consists of equations to predict annual BMI gain by age, sex and BMI, BMI was predicted annually for each child in the intervention cohort and the control cohort until age 15 years. At each annual cycle, BMI was converted to weight status (healthy weight, overweight or obesity) using WHO growth standards ^26^. Utility weights were then applied at each cycle, and for each participant, based on weight status. The values applied are shown in Table S4a and were derived from meta-analysis of 11 studies reporting utility values by weight status ^27^. Similarly, at each cycle, we assigned healthcare costs accumulated over a year. These healthcare costs account for government funded hospital costs and non-hospital medical costs (primary and specialist care, medicines and tests). As in another cost-effectiveness evaluation ^28^ using the same modelling methods, we used a top-down approach to estimate these costs based on administrative data on hospital costs published by the Australian Institute of Health and Welfare (AIHW), non-hospital costs from Medicare statistics ^29^, estimates of excess costs of overweight and obesity compared to health weight ^30^ and age-specific estimates of prevalence of overweight and obesity from the Australian National health Survey ^31^. The relevant estimates and attributed healthcare costs are presented in table S4b.

For each participant in intervention and control cohorts, total QALYs and total healthcare costs were calculated by summing the annual utilities and healthcare costs over the modelled timeframe using an annual 5% discount rate (3% in sensitivity analysis 4).

After incorporating intervention costs, the modelled BMI, total QALYs and total healthcare costs for the intervention and control were used to calculate the point estimates for cost-effectiveness and cost-utility ICERs. To evaluate uncertainty in these estimates, we took 1000 bootstrapped samples (with replacement) from each cohort to calculate 1000 ICERs. The 95% confidence intervals of the ICERs were determined by taking the 2.5^th^ and 97.5^th^ percentiles of these bootstrapped ICERs.

**Table S4a:** Utility values attributed to each weight status group

| Healthy | Overweight | Obese |
| --- | --- | --- |
| 0.85 | 0.83 | 0.82 |
|  |  |  |
|  |  |  |

**Table S4b: Age-specific healthcare costs attributed to each weight status group**

| Age group (years) | Relative cost compared to healthy weight | | | Annual Hospital Costs (AUD) | | | Mean annual hospital costs (AUD) | Annual Medicare costs (AUD) | | | Mean annual Medicare costs (AUD) |
| --- | --- | --- | --- | --- | --- | --- | --- | --- | --- | --- | --- |
|  | Healthy | Overweight | Obese | Healthy | Overweight | Obese |  | Healthy | Overweight | Obese |  |
| 5-9 | 1 | 1.12 | 1.23 | 382.98 | 428.94 | 471.07 | 403.03 | 298.86 | 334.72 | 367.60 | 326.40 |
| 10-14 |  |  |  | 425.79 | 476.89 | 523.73 | 449.53 | 293.91 | 329.18 | 361.51 | 335.40 |
| 15-19 |  |  |  | 771.84 | 864.46 | 949.36 | 814.43 | 401.07 | 449.19 | 493.31 | 445.40 |

**Appendix 5 – Detailed Romp & Chomp intervention costs using high cost estimates**

| Parameters | | Mean values | Assumptions | Data source |
| --- | --- | --- | --- | --- |
| Time cost | | | | |
| Project Administrators | | As per base case analysis |  |  |
| Health Promotion Officers | | AUD160,712,555 | Assumed 1 Health Promotion Officer (1 FTE) per LGA (n=562) ^7^ | “Other Health Diagnostic and Promotion Professionals” fulltime weekly salary ^4-6^, including 14.5% on-costs and 17.5% leave loading ^6^ |
| Web Support Technician | | As per base case analysis |  |  |
| Early childhood carers | | AUD176,244 | Assumed 1 hour training and 1 hour of sweet drink demonstration performance for 1 Child Carer per FDC (n=906) ^3,20^ | “Child Carers” hourly rate ^5,6^, including 14.5% on-costs and 17.5% leave loading ^6^ |
| Early childhood educators | | AUD4,267,137 | Assumed 1 hour training and 1 hour of sweet drink demonstration performance for 1 Child Carer per LDC and preschool (n=11,557) ^3^ | “School Teachers” hourly rate ^5,6^, including 14.5% on-costs and 17.5% leave loading ^6^ |
| ECEC managers | | As per base case analysis |  |  |
| Dentist | | AUD3,108,970 | Assumed 1.5 hour engaging with parents and early childhood carers and educators training (n=12,463) ^3,20^ | “Health therapy professional” hourly rate ^5,6^, including 14.5% on-costs and 17.5% leave loading ^6^ |
| Travel cost | | | | |
| Health Promotion Officers travel to attend festivals | | AUD267,519 | Assumed 4 festival presences within each LGA, 31.2 kilometres each 1-way trip to festival locations, based on highest average commuting distance to Place of Work by Greater Capital City Statistical Area ^32^ | Car expenses, Australian Taxation Office ^9^ |
| Health Promotion Officers travel to provide training session for FDC, LDC and preschool | | AUD1,483,138 | Assumed 31.2 kilometres each 1-way trip to FDC, LDC and preschool, based on highest average commuting distance to Place of Work by Greater Capital City Statistical Area ^32^ | Car expenses, Australian Taxation Office ^9^ |
| Material and equipment cost | | | | |
| Festival stall booking | | As per base case analysis |  |  |
| Resources for festival attendances | |  |  |  |
| Printed training booklets for Health Promotion Officers and early education and care settings | |  |  |  |
| Marketing and promotional materials for early education and care settings | |  |  |  |
|  | Active play program print | As per base case analysis |  |  |
|  | Active play poster |  |  |  |
|  | Daily Fruit & Veg poster |  |  |  |
|  | Daily Fruit & Veg fundraising fact sheet |  |  |  |
|  | Daily water poster |  |  |  |
|  | Fundraising ideas sticker |  |  |  |
|  | Energy dense snack display |  |  |  |
| Resources for “sweet drinks demonstrations” | | As per base case analysis |  |  |
| Marketing and promotional materials for participants | |  |  |  |
|  | Active play postcards | AUD154,530 | Assumed 1 unit of each marketing and promotion material type for each participant each year (n= 884,179) ^1-3^ | Market rates ^18^ |
|  | Why Fruit & Veg? postcards | AUD154,530 |  |  |
|  | Eat a Variety of Fruit & Veg postcards | AUD154,530 |  |  |
|  | Lunch Box & Snack Ideas postcards | AUD154,530 |  |  |
|  | Increase fruit & veg postcards | AUD154,530 |  |  |
|  | Increase fruit & veg stickers | AUD455,296 |  |  |
|  | Daily water brochures | AUD271,636 |  |  |
|  | Daily water postcards | AUD154,530 |  |  |
|  | Resource folders to families | AUD1,909,926 |  |  |
|  | "Tap into water" stickers | AUD455,296 |  |  |
|  | "No sweet drinks" tip sheet pads | AUD910,890 |  | Market rates ^16,21^ |
|  | Active play tip sheets | AUD910,890 |  | Market rates ^16,21^ |
| Web domain | | AUD1,166 |  | Market rates ^23^ |
| Web hosting | | AUD687 |  | Market rates ^23^ |
| *Kids – ‘Go for your life’* | | | | |
| Marketing and promotion materials for childcare settings | | | | |
|  | Practical activity sheet - playing alone | As per base case analysis |  |  |
|  | Practical activity sheet - playing with others |  |  |  |
| Marketing and promotional materials for participants | |  |  |  |
|  | Tap into water everyday – Newsletter | AUD1,821,780 | Assumed 1 unit of each marketing and promotion material type for each participant each year (n=884,179) ^1-3^ | Market rates ^16,21^ |
|  | Tap into water everyday – Message sheet | AUD154,530 |  | Market rates ^18^ |
|  | Tap into water everyday – Parent tip sheet - why no sweet drinks for children | AUD910,890 |  | Market rates ^16,21^ |
|  | Tap into water everyday – Parent tip sheet magnet - healthy family drink tips | AUD1,652,508 |  |  |
|  | Tap into water everyday – Drink bottle | As per base case analysis |  |  |
|  | Plant fruit and veg in your lunchbox – Newsletter | AUD3,643,560 |  | Market rates ^16,21^ |
|  | Plant fruit and veg in your lunchbox – Message sheet | AUD154,530 |  | Market rates ^18^ |
|  | Plant fruit and veg in your lunchbox – Parent tip sheet - 1-2 years, 3-5 years | AUD910,890 |  | Market rates ^16,21^ |
|  | Plant fruit and veg in your lunchbox – Parent tip sheet - Healthy lunch box | AUD910,890 |  |  |
|  | Plant fruit and veg in your lunchbox – Parent tip sheet - Food in the first year of life | AUD910,890 |  |  |
|  | Move, Play and Go – Newsletter | AUD3,643,560 |  |  |
|  | Move, Play and Go – Message sheet | AUD154,530 |  | Market rates ^18^ |
|  | Move, Play and Go – Parent tip sheet - Getting your family active | AUD910,890 |  | Market rates ^16,21^ |
|  | Move, Play and Go – Parent tip sheet - It's child's play | AUD910,890 |  |  |
|  | Move, Play and Go – Parent tip sheet - The hidden treasures of play | AUD910,890 |  |  |
|  | Move, Play and Go – Parent tip sheet - Rethinking outdoor play | AUD910,890 |  |  |
|  | Turn off, switch to play – Newsletter | AUD3,643,560 |  |  |
|  | Stride and ride – Message sheet | AUD154,530 |  | Market rates ^18^ |
| *Smiles 4 Miles* | | | | |
| Marketing and promotion materials for childcare settings | | | | |
|  | Oral health newsletters to childcare | As per base case analysis |  |  |
|  | Early childhood services toolkit |  |  |  |
|  | Drink well fact sheet |  |  |  |
|  | Eat well fact sheet |  |  |  |
|  | Clean well fact sheet |  |  |  |
|  | Play well fact sheet |  |  |  |
|  | Stay well fact sheet |  |  |  |
|  | Drink well tip card |  |  |  |
|  | Eat well tip card |  |  |  |
|  | Clean well tip card |  |  |  |
|  | Play well tip card |  |  |  |
|  | Stay well tip card |  |  |  |
| Marketing and promotional materials for participants | |  |  |  |
|  | Fact sheet - Looking after your child's month, teeth and gums | AUD910,890 | Assumed 1 unit of each marketing and promotion material type for each participant (n= 884,179) ^1-3^ | Market rates ^16,21^ |
|  | Drink well, eat well, clean well tip cards | AUD455,445 |  |  |
|  | Fact sheet - How to brush guide | AUD455,445 |  |  |
|  | Fact sheet - How to brush your child's teeth guide | AUD455,445 |  |  |
|  | Fact sheet - Tooth tips 1-12 months, 12-18 months, 18 months-6 years | AUD910,890 |  |  |
|  | Tip cards poster | AUD371,484 |  | Market rates ^18^ |
|  | Family information handout | AUD455,445 |  | Market rates ^16,21^ |
|  | Newsletters to families about healthy eating and oral health policy | AUD455,445 |  |  |
|  | Letter to families regarding policy feedback | AUD455,445 |  |  |
|  | Letter to families regarding the Drink well, Eat well survey | AUD455,445 |  |  |
|  | Letter to families regarding the Drink well, Eat well survey feedback | AUD910,890 |  |  |
|  | Family handout - How can children access dental services? | AUD455,445 |  |  |
|  | Letter to families about accessing public dental services | AUD455,445 |  |  |
|  | Family resources | AUD6,831,675 |  | Market rates ^16,21^ |
|  | Lunch boxes for participants | As per base case analysis |  |  |
| Estimated total intervention costs per year | | **AUD304,902,407** |  |  |
| Estimated total intervention costs per participant per year | | **AUD160** |  |  |

Notes: AUD: Australian dollar; FDC: family day care; FTE: full-time equivalent; ICT: Information and communications technology; KGFYL: Kids-Go for your life; LDC: long day care; LGA: local government area; S4M: Smiles 4 Miles

**Appendix 6 – Cost-effectiveness plane**

**Figure 1: Cost-effectiveness plane and cost-effectiveness acceptability curve – Sensitivity analysis 1**

Notes: AUD: Australian dollar; QALY: quality-adjusted life year

**Figure 2: Cost-effectiveness plane and cost-effectiveness acceptability curve – Sensitivity analysis 2**

Notes: AUD: Australian dollar; QALY: quality-adjusted life year

**Figure 3: Cost-effectiveness plane and cost-effectiveness acceptability curve – Sensitivity analysis 3**

Notes: AUD: Australian dollar; QALY: quality-adjusted life year

**Figure 4: Cost-effectiveness plane and cost-effectiveness acceptability curve – Sensitivity analysis 4**

Notes: AUD: Australian dollar; QALY: quality-adjusted life year

**Appendix 7 - Results comparison between different interventions**

| **Intervention** | **Brief intervention description** | **Target population** | **Country** | **Economic evaluation type** | **Perspective** | **Time horizon** | **Intervention cost per participant^#^** | **Effect inputs** | **Intervention effect** | **Long-term health outcomes** | **Results^#^** |
| --- | --- | --- | --- | --- | --- | --- | --- | --- | --- | --- | --- |
| R&C | Community-wide intervention to encourage healthy eating, physical activity and attainment of healthy weight | Children aged 0-5 years in ECEC settings | Australia | CEA, CUA, using the EPOCH model ^33^ | Funder perspective | 0-15 years | AUD93 | BMI | -0.06kg/m^2^ | QALY | CEA:  AUD1,126/BMI unit avoided  CUA:  AUD26,399/QALY gained |
| POI ^28^ | Childhood obesity prevention intervention via sleep, nutrition and physical activity interventions | Children aged 0-2 years | Australia | CEA, CUA, using the EPOCH model ^33^ | Health funder perspective | 0-15 years | POI Sleep arm: AUD184 | BMI | -0.26kg/m^2^ | QALY | CEA:  AUD558/BMI unit avoided  CUA:  AUD18,125/QALY gained |
|  |  |  |  |  |  |  | POI Combination arm:  AUD601 | BMI | -0.11kg/m^2^ | QALY | CEA:  AUD5,164/BMI unit avoided  CUA:  AUD94,667/QALY gained |
| A generic community-led intervention ^34^ | Community-led intervention, incorporating: (i) nutrition strategies in schools, (ii) physical activity strategies in schools, (iii) infrastructure change at school and (iv) changes within the community | Children and adolescents aged 5-18 years in primary and secondary schools | Australia | CUA, modelled | Limited societal perspective | Lifetime | AUD589 | BMI-z | -0.07 BMI-z | HALY | CUA:  AUD9,619/HALY gained |
| Be Active Eat Well ^35^ | Community-wide capacity-building program to promote healthy eating and physical activity | Children aged 5-12 years in primary schools | Australia | CEA, CUA, modelled | Societal perspective | Lifetime | AUD453 | BMI | -0.28kg/m^2^ | DALY | CEA:  AUD525/BMI unit avoided  CUA: AUD26,514/DALY saved |
| Project Energize ^36^ | Multicomponent school-based physical activity and nutrition program | Children aged 6-11 years | New Zealand | CUA, modelled | Funder perspective | Lifetime | AUD54 | BMI | Children aged 6-8 years:  -0.50kg/m^2^  Children aged 9-11 years:  -0.55kg/m^2^ | QALY | CUA:  Children aged 6-8 years: AUD27,171/QALY gained  Children aged 9-11 years: AUD29,260/QALY gained |
| APPLE ^37^ | School and community-wide environment changes via the provision of community activity coordinators and nutrition education | Children aged 5-12 years | New Zealand | CEA, CUA, within trial | Societal perspective | Four years | AUD1,591 | BMI-z | -0.18 at 2 years (the end of the intervention)  -0.17 at 4 years (at 2 year follow up) |  | CEA:  Children aged 13 years:  AUD825 per kg of weight-gain prevented  Children aged 7 years:  AUD2,121 per kg of weight-gain prevented  CUA:  No differences in health utilities index measures |

Notes: **^#^** All costs have been converted to 2018 Australian dollars using the purchasing power parities ^38^ and consumer price index ^39^. AUD: Australian dollars; BMI: body mass index; BMI-z: body mass index z-score; CEA: cost-effectiveness analysis; CUA: cost-utility analysis; DALY: disability-adjusted life year; HALY: health-adjusted life year; kg: kilogram; m^2^: meter square; NA: not available QALY: quality-adjusted life year

**REFERENCES**

1. Childhood Education and Care, Australia, June 2017. Australian Bureau of Statistics; 2018. h<ttps://www.abs.gov.au/AUSSTATS/abs@.nsf/Lookup/4402.0Main+Features1June%202017?OpenDocument.> Accessed 30 November.

2. Australian Bureau of Statistics. TABLE 59. Estimated Resident Population By Single Year Of Age, Australia In. Canberra2019.

3. Australian Bureau of Statistics. Preschool Education, Australia, 2018. Australian Bureau of Statistics,. h<ttps://www.abs.gov.au/ausstats/abs@.nsf/mf/4240.0.> Published 2019. Updated 13 February. Accessed 15 November, 2019.

4. Fair Word Ombudsman. Payment for Annual Leave. Australian Government. h<ttps://www.fairwork.gov.au/leave/annual-leave/payment-for-annual-leave.> Published 2019. Accessed 20 November, 2019.

5. Australian Bureau of Statistics. Employee Earnings and Hours, Australia, May 2018. Australian Bureau of Statistics,. h<ttps://www.abs.gov.au/ausstats/abs@.nsf/latestProducts/6306.0Media%20Release1May%202018.> Published 2019. Updated 22 January. Accessed 15 November, 2019.

6. Australian Bureau of Statistics. Labour Costs, Australia, 2015-16. h<ttps://www.abs.gov.au/AUSSTATS/abs@.nsf/mf/6348.0.> Published 2017. Updated 10 August. Accessed 10 November, 2019.

7. Australian Bureau of Statistics. Australian Statistical Geography Standard (ASGS): Volume 3 - Non ABS Structures, July 2019. Australian Bureau of Statistics,. h<ttps://www.abs.gov.au/ausstats/abs@.nsf/Lookup/by%20Subject/1270.0.55.003~July%202019~Main%20Features~Local%20Government%20Areas%20(LGAs)~2.> Published 2019. Updated 31 July. Accessed 15 November, 2019.

8. Australian Bureau of Statistics. 2071.0.55.001 - Census of Population and Housing: Commuting to Work - More Stories from the Census, 2016 h<ttps://www.abs.gov.au/ausstats/abs@.nsf/Lookup/by%20Subject/2071.0.55.001~2016~Main%20Features~Commuting%20Distance%20for%20Australia~1#:~:text=In%20Australia%2C%20the%20average%20commuting,than%2020%20km%20to%20work.> Published 2018. Updated 22 May. Accessed 1 August, 2020.

9. Australian Taxation Office. Car expenses. Australian Taxation Office. h<ttps://www.ato.gov.au/Individuals/Income-and-deductions/Deductions-you-can-claim/Vehicle-and-travel-expenses/Car-expenses/.> Published 2019. Accessed 25 November, 2019.

10. Festival of the Sea. Festival of the Sea Barwon Heads. h<ttps://www.festivalofthesea.com.au/.> Published 2020. Accessed 15 June, 2020.

11. Markets and Fairs. Cronulla Easter Market. h<ttps://www.marketsandfairs.com.au/Event/CRONULLA-EASTER-MARKET-Cambridge-Markets-Postponed-to-23rd-August.> Published 2020. Accessed 15 June, 2020.

12. Springwood Foundation Day Festival. Market Stall Holders. h<ttps://www.foundationday.org.au/index.php/join-in/stalls.> Published 2020. Accessed 16 June, 2020.

13. Woodford Folk Festival. Woodford Folk Festival Stall Fees h<ttps://woodfordfolkfestival.com/get-involved/stallholders/woodford-folk-festival-stall-fees/.> Published 2020. Accessed 17 June, 2020.

14. Rotary Club of Eltham. Stallholder Information. h<ttps://elthamfestival.org.au/stallholders-information/.> Published 2020. Accessed 25 June, 2020.

15. The CQUniversity. Village Festival. h<ttps://thevillagefestival.org.au/wp-content/uploads/2019/03/Stallholders-Guide-2019_-Final.pdf.> Published 2019. Accessed 17 June, 2020.

16. Officerworks. Officeworks. h<ttps://www.officeworks.com.au/.> Published 2019. Accessed 20 November, 2019.

17. Kmart. Kmart. h<ttps://www.kmart.com.au/.> Published 2019. Accessed 20 November, 2019.

18. vistaprint. vistaprint. h<ttps://www.vistaprint.com.au/?GP=12%2f11%2f2019+23%3a11%3a38&GPS=5563764191&GNF=1.> Published 2019. Accessed 25 November, 2019.

19. Woolworths. Woolworths. h<ttps://www.woolworths.com.au/.> Published 2019. Accessed 25 November, 2019.

20. Department of Education. Child Care in Australia report Financial year 2018-19. h<ttps://education.govcms.gov.au/child-care-australia-report-financial-year-2018-19.> Published 2019. Updated 25 October. Accessed 15 November, 2019.

21. Deakin University. Change in printing costs. h<ttps://blogs.deakin.edu.au/deakinlife/2019/08/06/change-in-printing-costs/.> Published 2019. Updated 6 August. Accessed 24 October, 2019.

22. Coles. Coles. h<ttps://www.coles.com.au/.> Published 2019. Accessed 25 November, 2019.

23. GoDaddy. GoDaddy. h<ttps://au.godaddy.com/.> Published 2020. Accessed 6 February, 2020.

24. Australian Demographic Statistics, Mar 2019. Australian Bureau of Statistics,; 2019. h<ttps://www.abs.gov.au/AUSSTATS/abs@.nsf/DetailsPage/3101.0Mar%202019?OpenDocument.> Accessed 30 November.

25. Hayes A, Tan EJ, Lung T, Brown V, Moodie M, Baur L. A New Model for Evaluation of Interventions to Prevent Obesity in Early Childhood. 2019;10(132).

26. WHO Multicentre Growth Reference Study Group. WHO Child Growth Standards based on length/height, weight and age. *Acta paediatrica (Oslo, Norway: 1992) Supplement.* 2006;450:76.

27. Brown V, Tan E, Hayes A, Petrou S, Moodie ML. Utility values for childhood obesity interventions: a systematic review and meta‐analysis of the evidence for use in economic evaluation. *Obesity reviews.* 2018;19(7):905-916.

28. Tan EJ, Taylor RW, Taylor BJ, Brown V, Hayes AJ. Cost-Effectiveness of a Novel Sleep Intervention in Infancy to Prevent Overweight in Childhood. *Obesity.* 2020;n/a(n/a).

29. The Department of Health. *Annual Medicare Statistics - Financial Year 1984-85 to 2017-18.* Canberra: The Department of Health;2018.

30. Clifford SA, Gold L, Mensah FK, et al. Health‐care costs of underweight, overweight and obesity: Australian population‐based study. *Journal of paediatrics and child health.* 2015;51(12):1199-1206.

31. Australian Bureau of Statistics. National Health Survey: First Results, 2014-15. h<ttp://www.abs.gov.au/ausstats/abs@.nsf/mf/4364.0.55.001.> Published 2015. Accessed 20 September, 2020.

32. Bureau of Infrastructure Transport and Regional Economics (BITRE). Australia’s commuting distance: cities and regions. In: Development DoIaR, ed. Canberra: Commonwealth of Australia; 2015.

33. Brown V, Ananthapavan J, Sonntag D, Tan EJ, Hayes A, Moodie M. The potential for long-term cost-effectiveness of obesity prevention interventions in the early years of life. 2019;14(8):e12517.

34. Ananthapavan J, Nguyen PK, Bowe SJ, et al. Cost-effectiveness of community-based childhood obesity prevention interventions in Australia. *International Journal of Obesity.* 2019;43(5):1102.

35. Moodie ML, Herbert JK, de Silva‐Sanigorski AM, et al. The cost‐effectiveness of a successful community‐based obesity prevention program: The be active eat well program. 2013;21(10):2072-2080.

36. Rush E, Obolonkin V, McLennan S, et al. Lifetime cost effectiveness of a through-school nutrition and physical programme: Project Energize. *Obesity research & clinical practice.* 2014;8(2):e115-e122.

37. McAuley KA, Taylor RW, Farmer VL, et al. Economic evaluation of a community‐based obesity prevention program in children: the APPLE project. 2010;18(1):131-136.

38. Purchasing power parities (PPP). 2017. h<ttps://www.oecd-ilibrary.org/content/data/1290ee5a-en.>

39. Australian Bureau of Statistics. Consumer Price Index, Australia, Sep 2019. Australian Bureau of Statistics,. <https://www.abs.gov.au/AUSSTATS/abs@.nsf/allprimarymainfeatures/938DA570A34A8EDACA2568A900139350?opendocument>. Published 2019. Updated 30 October. Accessed 15 November, 2019.
